# Christian Science

**Published:** 1900-06

**Authors:** 


Christian Science. An Exposition of Mrs Eddy's Wonderful Discovery, Including its Legal Aspects. A Plea for Children and Other Helpless Sick. By William A. Purrington, Lecturer in the University and Bellevue Hospital Medical College, and in the New York College of Dentistry, upon Law in Relation to Medical Practice; one of the authors of A System of Legal Medicine. New York: E. B. Treat & Co. 1900.

This interesting book comes when called, and gives timely and reliable information upon an important and prominent topic. We consider it good fortune that its author is neither a teacher of Christianity nor practitioner of medicine; being neither the one nor the other, but a student and teacher of law, he can the more appropriately defend Christianity and medicine against the common enemy.

Those of us who are willing to take impressions at second-hand—and many must do this from force of circumstances—will find in chapters I. and II. a fair and reliable illustration of what Eddyism claims to be, and also how far short of this claim the author finds its performance; these chapters are critical, do not assume to be sympathetic or friendly, but are fair and reliable.

We wish the author could have taken the time to write, as he is so well fitted to write, an admonition as to what the law may not do to the disciples of Eddyism. Our fathers thought by means of statutes, codes of ethics, by-laws and the like, to prevent our people from experimenting with homeopathy—with the result of making homeopathy live and flourish for a century. Undoubtedly the same or similarly unwise repressive treatment of Eddyism would be followed by the same uncalled for and confounding contribution to the growth and spread of the contagion. Warning and admonition against this treatment of error in religion or medical thought, would come with singular appropriateness from the legal profession.

